# Potential of using facial thermal imaging in patient triage of flu-like syndrome during the COVID-19 pandemic crisis

**DOI:** 10.1371/journal.pone.0279930

**Published:** 2023-01-18

**Authors:** Ana Carolina Makino Antunes, Alexandre Aldred, Gabriela Pinheiro Tirado Moreno, João Alberto de Souza Ribeiro, Paulo Eduardo Brandão, Gisely Toledo Barone, Juliana de Amorin Conselheiro, Alessandra C. Goulart, Ivan Cesar Desuó, Guilherme Gomes

**Affiliations:** 1 Department of General Surgery, Hospital Universitário, Universidade de São Paulo, São Paulo, São Paulo, Brasil; 2 Predikta Soluções em Pesquisa Ltda, São Paulo, São Paulo, Brasil; 3 Department of Internal Medicine, The Veterinary Teaching Hospital, School of Veterinary Medicine and Animal Science of University of São Paulo, São Paulo, São Paulo, Brasil; 4 Termodiagnose Institute, São Paulo, São Paulo, Brasil; 5 Department of Preventive Veterinary Medicine and Animal Health, School of Veterinary Medicine, University of São Paulo, São Paulo, São Paulo, Brasil; 6 Center for Clinical and Epidemiological Research, Hospital Universitário, Universidade de São Paulo, São Paulo, São Paulo, Brasil; 7 Department of Internal Medicine, Hospital Universitário, Universidade de São Paulo, São Paulo, São Paulo, Brasil; FDA: US Food and Drug Administration, UNITED STATES

## Abstract

The screening of flu-like syndrome is difficult due to nonspecific symptoms or even oligosymptomatic presentation and became even more complex during the Covid-19 pandemic. However, an efficient screening tool plays an important role in the control of highly contagious diseases, allowing more efficient medical-epidemiological approaches and rational management of global health resources. Infrared thermography is a technique sensitive to small alterations in the skin temperature which may be related to early signs of inflammation and thus being relevant in the detection of infectious diseases. Thus, the objective of this study was to evaluate the potential of facial thermal profiles as a risk evaluator of symptoms and signs of SARs diseases, using COVID-19 as background disease. A total of 136 patients were inquired about the most common symptoms of COVID-19 infection and were submitted to an infrared image scanning, where the temperatures of 10 parameters from different regions of the face were captured. We used RT-qPCR as the ground truth to compare with the thermal parameters, in order to evaluate the performance of infrared imaging in COVID-19 screening. Only 16% of infected patients had fever at the hospital admission, and most infrared thermal variables presented values of temperature significantly higher in infected patients. The maximum eye temperature (MaxE) showed the highest predictive value at a cut-off of >35.9°C (sn = 71.87%, sp = 86.11%, LR+ = 5.18, LR- = 0.33, AUC = 0.850, p < 0.001). Our predictive model reached an accuracy of 86% for disease detection, indicating that facial infrared thermal scanning, based on the combination of different facial regions and the thermal profile of the face, has potential to act as a more accurate diagnostic support method for early COVID-19 screening, when compared to classical infrared methods, based on a single spot with the maximum skin temperature of the face.

## 1. Introduction

The COVID-19 pandemic has been shaking the world victimizing more people every day. The current data shows that there are more than 525 million infected and more than 6 million deaths [1]. In Brazil, the number of confirmed cases has surpassed 30 million, and more than 660 thousand deaths were reported, which puts Brazil in third place in numbers of deaths, behind the USA and India [1].

The clinical presentation of COVID-19 varies from asymptomatic infection to a severe respiratory failure [2–4], hence efforts to provide support for a quick screening to help diagnose the disease, ensuring proper treatment for the patients. Testing for COVID-19 using RT-qPCR (real-time quantitative reverse-transcriptase polymerase chain reaction) has been widely adopted, and despite its high accuracy, it remains expensive and has a laboratory turnaround time of 24 to 48 hrs [5]. In this context, screening centers, walk-in urgent care clinics, and Intensive Care Units (ICUs) have critical importance during a pandemic—while the first ones allow effective initial triage and risk evaluation, the last one provides the necessary equipment and protocols to maintain the clinical stability of critically ill patients, allowing them to recover from their illness. In these centers, decision-making must be quick and assertive, based on multiple health parameters that allow critical resource management.

Regarding the novel coronavirus disease, fever had been described as the most common clinical finding, leading to health centers, offices, and airports to invest in the technology of mass screening for this particular parameter, usually through the skin temperature of the forehead as an indication of core body temperature, however, this approach has serious limitations, low sensitivity and should be avoided [6, 7]. Wang et al. [8] found that infrared thermography can provide superior performance for measuring elevated body temperature than conventional NCITs devices, partly due to IRT measurements having more pixels per area and thus being more thermally stable. The authors also provided a set of calibration methods in order to enhance IRT clinical accuracy and confirmed that temperature of inner canthi or full-face max temperature provided the highest performance to evaluate body temperature.

Fixed infrared thermography proved to be effective for identification of subjects with influenza-like illness from febrile patterns; Hinnerichs [9] implemented a surveillance tool on United States Navy and Marine vessels and found that IRT could differentiate afebrile from febrile in 91% of the time. However, beyond the capacity of act as a thermometer to detect elevated body temperature, infrared thermography (IRT) can also be used as a diagnostic support tool to capture early signs and symptoms of infectious diseases, using different regions of interest and/or thermal asymmetries. This approach is still strikingly limited and still poorly explored, a few studies just begin to evaluate its potential, with encouraging results [10, 11]. IRT allows, through a thermal image produced by a sensor that captures infrared, to indirectly evaluate the metabolism and physiology of the individual, in a non-invasive way, without physical contact and in real-time with a fast turnaround response. This technique is remarkably sensible to detect small changes in the temperature of the skin, related mainly to alteration in the blood flow due to vasodilation or vasoconstriction [11, 12]. Such features of IRT may be especially important for detecting early signs of inflammation in the respiratory airways, usually associated with symptoms of infectious diseases such as COVID-19 and thus impacting the thermal profile of the subject, making this technique a potentially important screening tool.

Thus, this study aimed to evaluate the potential of infrared thermography to evaluate early signs and symptoms, through thermal images of different facial regions of subjects, and thus to analyze its thermal profile among the regions to be used as a support diagnostic tool in the screening for SARs using the COVID-19 as background disease, during the peak of the pandemic in 2020 in Brazil.

## 2. Methods

### 2.1. Population, participants and study design

This is a cross-sectional study conducted among patients (18 years or older) with flu symptoms or severe acute respiratory syndrome (SARS) suggestive of COVID-19 seen at the emergency department (ED) in the *Hospital Universitário* of the University of São Paulo (HU-USP). The HU-USP is a secondary community hospital located in the Butantan district, a low-middle income area with approximately 500,000 inhabitants on the west side of the city.

A total of 136 patients seen at the HU-USP emergency medical service from June to September 2020 met the inclusion and exclusion criteria, as described in this study. To search for a pathological pattern for this disease, only patients tested for SARS-CoV-2 RT-qPCR [13] had their data collected and analyzed. Patients with reduced mobility who were unable to remain seated long enough to acquire facial images (intubated patients, for example) and those who had not been tested for COVID-19 (RT-qPCR) were excluded from our sample. The thermal images were obtained in an evaluation room that has temperature and humidity control (temp: 22.2 ± 0.8°C, RH: 60.5±2.6% and dew point: 14.1±1.5°C).

Written informed consent was obtained from all participants or their guardians (mostly a close family member). The study was approved by the Research Ethics Committee of HU-USP, São Paulo, Brazil and is approved in the Plataforma Brasil system under CAAE—33665920.0.0000.0076. The personal data of all participants are protected by anonymity and the eventual use of thermal images of the subjects’ faces in scientific publications is covered by the consent form. The study report is in accordance with the STARD general guidelines for diagnostic accuracy studies [14].

### 2.2. Digital infrared thermal imaging (IRT) and data collection

To obtain the infrared parameters, we used a Flir T530 thermal camera (minimum resolution 320X240, accuracy ±2°C, thermal sensitivity less than 40 mK, 24° FOV, coupled with an automated analysis software).

All individuals with the flu-like syndrome had the previous acclimatization during hospital registration, and soon afterwards the thermal data collection started. The seated patient positioned himself 80 cm from the infrared camera, removed his protection mask, and remained practically still for 30s looking into the lens of the equipment. After this protocol, we obtained a sequence of radiometric filming of the subject face at 30 fps with a 30 second duration. Thermal images were taken and the infrared parameters were evaluated by a researcher blinded to the clinical data and RT-qPCR results. We used the thermal image to capture the thermal profile of the individual’s face, due to the limitations of infrared sensors to obtain the real or body temperature: ±2°C accuracy of the equipment, the influence of the sensor temperature, the difference between the nuclear/body temperature with the individual’s surface temperature and evaporative cooling in the face. Thus, our main objective was to extract thermal profiles to act as physiological markers, through different facial regions of interest (ROIs) between infected and non-contaminated people. In this context, we could obtain the highest potential of the thermal image and not just a point value similar to a non-contact thermometer. The thermal profile is a relationship among more than 70,000 temperature points, offering countless functional/physiological information not yet well explored in the health area.

The standard method used in the routine hospital for fever evaluation is based on contact body thermometry. For that, the medical staff commonly use a clinical mercury thermometer in the axilla of the patient with visual readout by staff during intake evaluation. As a threshold to indicate febrile state, we considered patients with axillary temperatures (clinical mercury thermometry) above 37.5°C as febrile.

In order to quantify and compare the thermal profile of the patients using infrared thermography, we extracted temperature values from different regions of interest of the face of each subject, as follows: TIC (max temperature of eyes inner canthi); EyeR (maximum temperature of right eye surface); EyeL (max temperature of left eye surface); LFR (max temperature of the right lateral portion of the nose surface); LFL (max temperature of left lateral of the nose); Nose (mean temperature of the nose surface); Mouth (mean temperature of the mouth); mE (mean temperature of both eyes); mL (mean temperature between both lateral portions of the nose); MaxE (max temperature between both eyes) and MaxL (max temperature between both sides of the nose).

Patients were analyzed in two groups: positive (PCR+) and negative (PCR-) for SARS-CoV 2 virus infection. Obtaining clinical data and thermal images of the volunteers’ faces was done before the collection by nasal/oropharyngeal swab of the secretion sample for analysis by RT-qPCR.

### 2.3. Data analysis

According to the distribution of continuous variables, data were presented as mean (standard deviation, SD) or median (interquartile range, IQR) and, both clinical and IRT parameters were compared between infected (PCR+) and non-infected (PCR-) using *T-test* for independent samples or Mann-Whitney U test.

To measure the diagnostic potential of each infrared variables for detecting COVID-19, a receiver operating curve was performed and the values of Sensitivity (Sn), Specificity (Sp), area under the curve (AUC), positive predictive value (PPV), negative predictive value (NPV), positive and negative likelihood ratios (LR+ and LR-) and cut-offs were calculated to provide validation for the diagnostic value of clinical and IRT parameters tested. We considered values of 0.5 for AUC as poor discrimination, 0.7 to 0.8 as fair, 0.8 to 0.9 as excellent, and more than 0.9 as outstanding [15, 16].

Finally, we build a classification model based on Random Forest (RF) algorithm to evaluate the potential of COVID-19 detection, considering all infrared ROIs (except TIC) as predictors and compared the results with the standard method, usually associated with infrared screening for infectious diseases; based on a single spot temperature of the eye inner canthus (in this paper, represented by TIC). For the RF model, we used cross validation with bootstrap resampling (K = 25) in order to tune and check model stability. Initially, the data was split 80/20 in train (108; 57 negatives and 51 positives) and test (28; 15 negatives and 13 positives). For model specification we used the “ranger” algorithm, which is part of the available options in the R package Tidymodels [17]. We fine tuned our model using a grid to optimize the RF hyperparameters, such as mtry (number of predictors that will be randomly sampled at each split when creating the tree models) and min_n (minimum number of data points in a node that is required for the node to split further), the number of trees was set to 1000, and the best validation split was chosen based on the highest value of AUC (S1 Fig). The workflow described above was based on [17, 18].

For model performance evaluation, we used the following metrics: sensitivity($\frac{True\;Positive}{True\;Positive+False\;Positive}$), specificity ($\frac{False\;Negative}{True\;Negative+False\;Positive}$), accuracy($\frac{Number\;of\;correct\;predictions}{Total\;Number\;of\;predictions}\times 100$) and AUC (Area under the curve).

As a reference standard, we used the RT-qPCR test for SARS-CoV-2 virus detection since it is still considered the gold standard test for the diagnosis of the disease and is also the common practice adopted in hospital and emergency medical services for cases suggestive of COVID-19. We used an overall significance index of 5% and all tests were performed using R v.4.1.2 [19]. No missing data were detected, nor indeterminate reference standard results were reported.

## 3. Results

**Fig 1:**
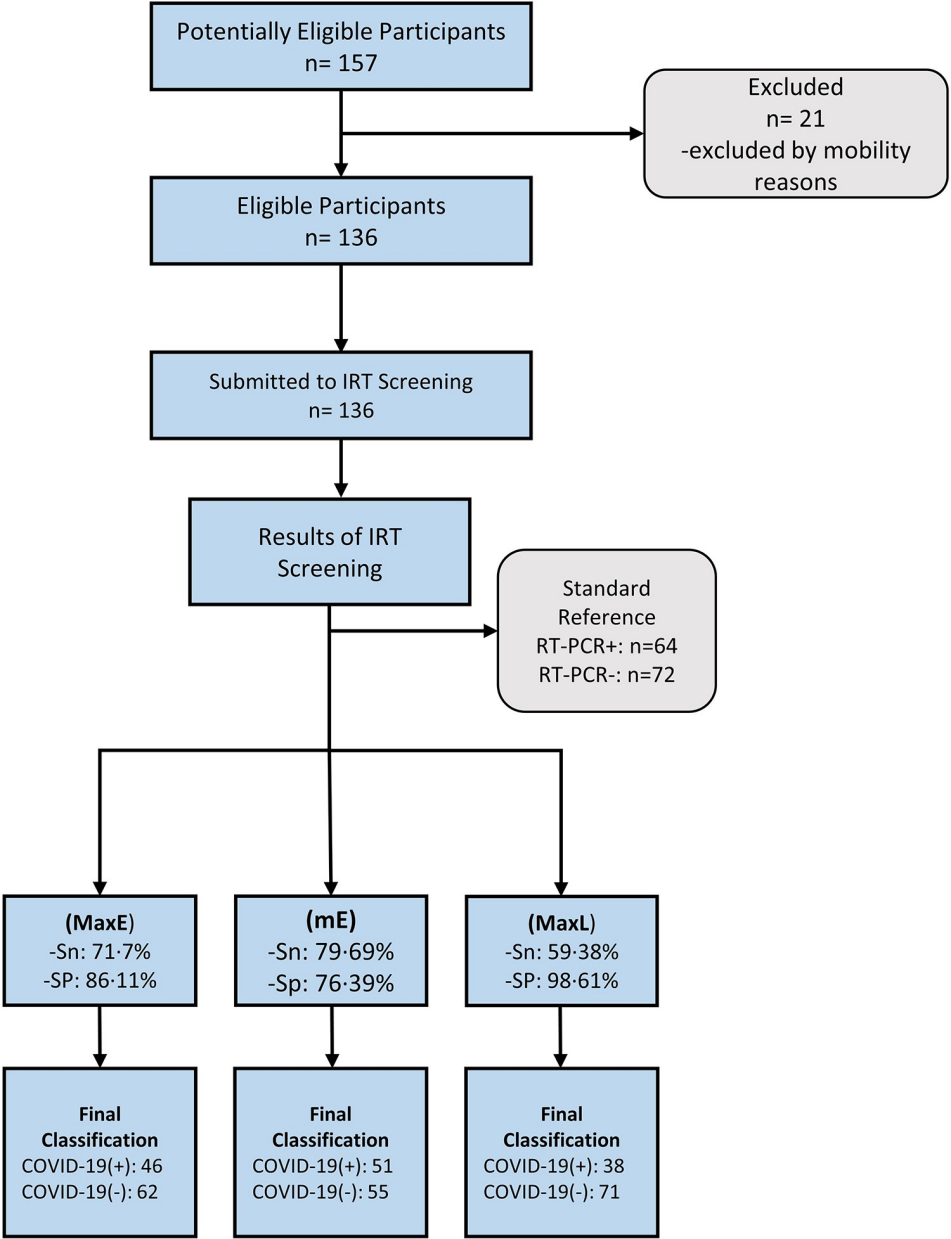
Flow chart diagram of the study design. Best infrared parameters: MaxE (maximum temperature of the Eyes); mE (mean temperature between the eyes); MaxL (maximum temperature of the lateral of the nose).

Among 136 patients (mean age: 49.88*±*17.84, 57% women) who sought care at the HU-USP with flu-like symptoms, 64 (47%) tested positive and 72 (53%) tested negative for the SARS-CoV-2 infection (RT-qPCR detection). A flow chart of the study design and the most important results can be seen in Fig 1. More details on the profile of the patients’ demographic characteristics of this study can be seen in Table 1.

**Table 1:** Demographic and symptom profiles of subjects. Symptoms with significant statistics differences between infected and non-infected for COVID-19 are in bold.

	PCR+	PCR-	p^#^
**Sex N(%)**			
M	30(47)	25(35)	0.12
F	34(53)	47(65)	
**Age (in years)**	51.70±16.42	46.78±17.95	0.10*
**Age Range N(%)**			
0–20	0(0)	3(4)	-
20–40	15(23)	23(32)	
40–60	26(41)	28(39)	
60>	23(36)	18(25)	
**Symptoms N(%)**			
**Fever**	**36(56)**	**26(36)**	**0.03**
Cough	38(59)	51(57)	0.80
Sore throat	22(34)	25(35)	0.97
Coryza	20(31)	30(42)	0.27
Dyspnea	23(36)	18(25)	0.14
Myalgia	28(44)	30(42)	0.72
**Anosmia**	**33(52)**	**17(24)**	**<0.001**
**Diarrhea / Emesis**	**18(28)**	**5(7)**	**<0.01**
Headache	15(23)	15(21)	0.67
**Onset to symptoms (in days)**	3 IQR 5	2 IQR 7	0.10
**Comorbidities N(%)**	20(13)	14(10)	0.32
Hipertension	7(11)	4(6)	-
Diabetes	3(5)	3(4)	
Dyslipidemia	1(2)	2(3)	
Asthma	4(6)	1(1)	
Chronic Obtrusive Pulmonary disease	2(3)	1(1)	
Smoking	0	4(6)	
Chronic heart disease	0	1(1)	
Obesity	0	1(1)	
Atrial Fibrillation	0	1(1)	
Chronic myeloid leukemia	1(2)	0	
Alzheimer	1(2)	0	

^#^p-values for all other signs were obtained from Mann-Whitney rank test for independent samples

*p-values for means comparisons were obtained from the t-student test for independent samples.

The most common symptoms reported by the patients were: fever, cough, sore throat, coryza, dyspnea, myalgia, anosmia, diarrhea/emesis, and headache. Only 2% of patients were asymptomatic at admission. Among the symptoms reported by patients, only fever (56% vs. 36%, p = 0.03), anosmia (52% vs. 24%, p<0.001) and diarrhea/emesis (28% vs. 7%, p<0.01) were statistically significant between infected (PCR+) and noninfected (PCR-) (Table 1). Regarding the onset of symptoms in days, no statistical difference was found between infected and non-infected (median: 3, 3 IQR 5 vs. median: 4.5, 2 IQR 7; p = 0.10). Only 16% of our sample had a body temperature equal to or greater than 37°C in the admission, with 45% of them having tested negative for COVID-19.

**Fig 2:**
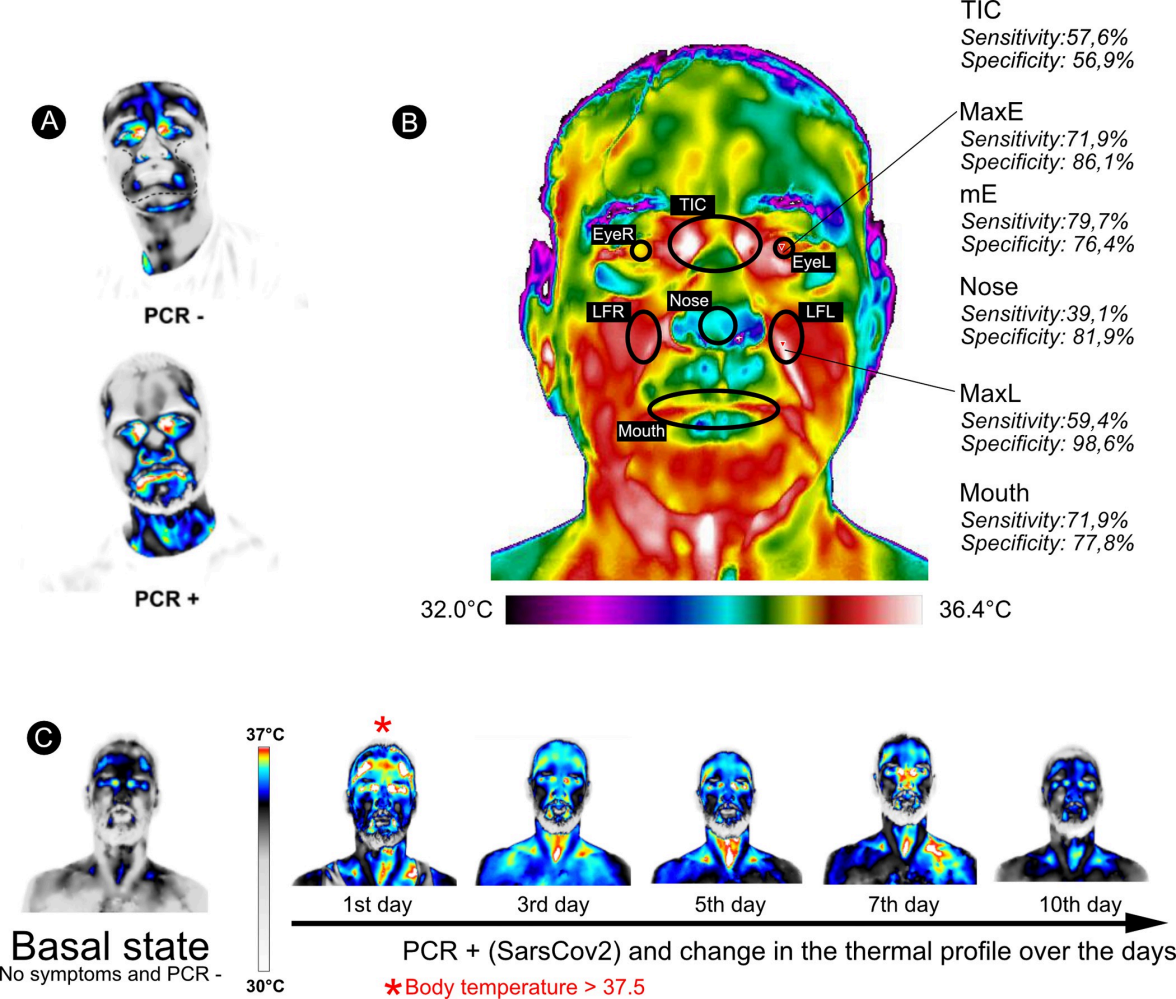
Thermal image profile of the analyzed patients’ faces. (A) The dotted line describes the region with the greatest significant difference between patients tested positive (PCR+) and negative (PCR-) for COVID-19; (B) Regions with greater sensitivity and specificity in the evaluation of the thermal image: MaxE (maximum temperature of the Eyes), MaxL (maximum temperature of the lateral of the nose), mE (mean temperature between the eyes), Nose (mean temperature of the nose surface), Mouth (mean temperature of the mouth) and (C) Evolution of facial thermal profile of a subject during the infection period (according to sequential PCR results), showing that IRT captured fever (T > 37.5°C) only in the first day of infection.

**Fig 3:**
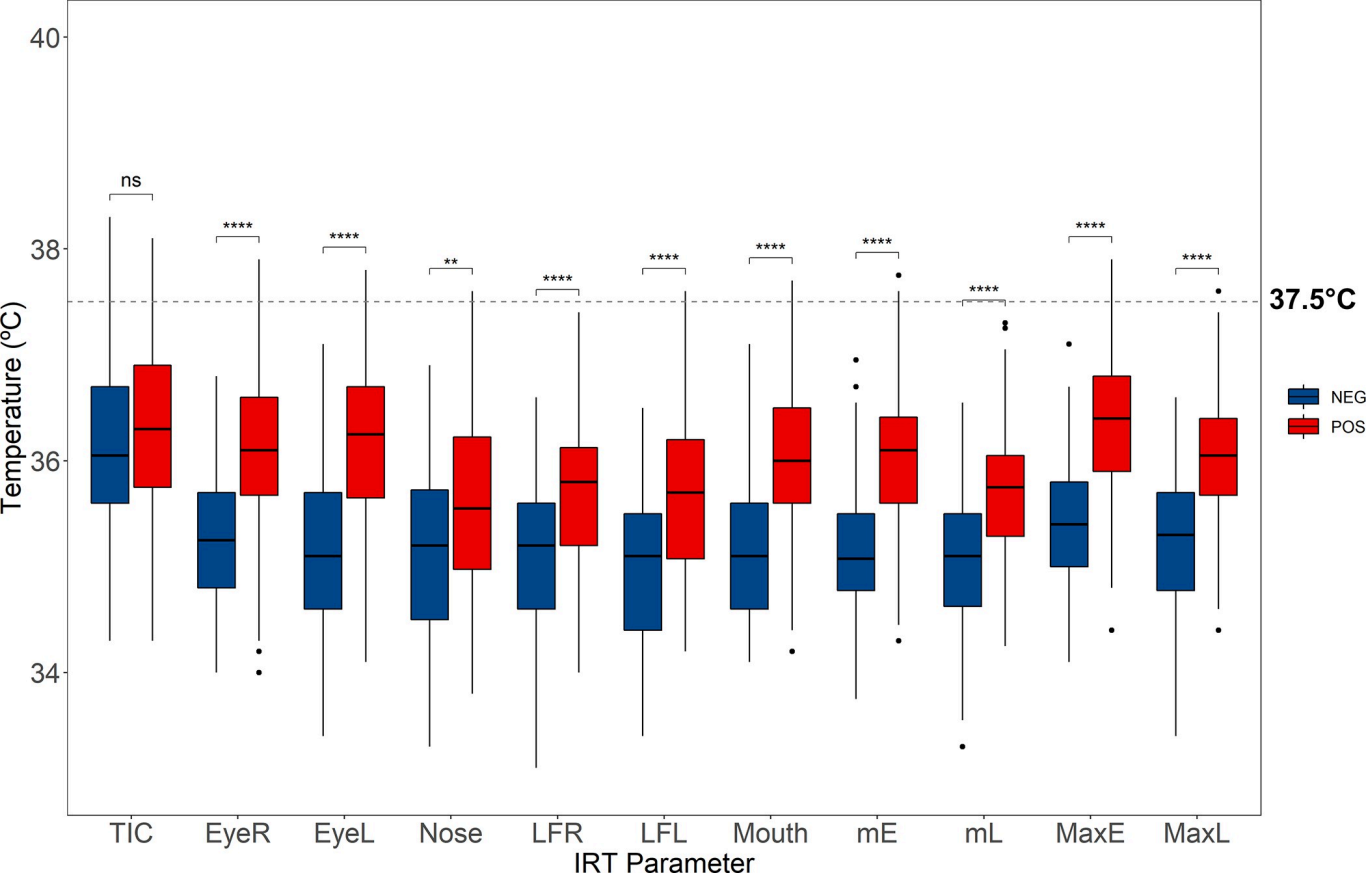
Boxplots of the IRT parameters between PCR+ and PCR-. The means were significantly higher for all IRT parameters, except TIC and Nose (see Table 2 for t-test results). Infected patients presented an overall higher face temperature then non-infected, however rarely within fever state (dashed line represents the temperature of 37.5°C). **** p<0.0001; ns–no statistical significance.

The thermal profile of the face of infected patients (PCR+) demonstrated a noticeable different pattern from the non-infected (PCR-), which seems to be well associated with one of the most common symptoms of SARs [11] related to upper airways inflammation, as can be seen in the regions of greatest heat dissipation in Fig 2. Except for TIC (t = -1.51, p = 0.132), all other infrared parameters were significantly higher in infected patients compared to the non-infected, as shown in Fig 3 and Table 2. It is important to note that, although the face of infected patients is generally warmer than those noninfected, the temperature range is rarely within the febrile state (Fig 3).

**Table 2:** Means±SD, t-test results and ROC analysis results between infected (PCR+) and non-infected (PCR-) patients for COVID-19. Parameters with best performance based on AUC comparisons after ROC analysis are presented in bold.

	Mean±SD		t-test		ROC Analysis			
	PCR+	PCR-	t-value	p*	AUC^#^	p	95% CI	
EyeR	36.03±0.88	35.23±0.66	-5.94	<0.001	0.781	<0.001	0.702	0.848
EyeL	35.59±0.90	35.15±0.74	-7,01	<0.001	0.803	<0.001	0.726	0.866
LFR	35.69±0.73	35.01±0.72	-5,39	<0.001	0.743	<0.001	0.661	0.814
LFL	35.69±0.79	34.99±0.67	-5,57	<0.001	0.751	<0.001	0.669	0.821
**MaxE**	**36.32±0.68**	**35.41±0.59**	**-8,31**	**<0.001**	**0.850**	**<0.001**	**0.779**	**0.906**
**MaxL**	**36.00±0.67**	**35.22±0.64**	**-6,88**	**<0.001**	**0.811**	**<0.001**	**0.735**	**0.873**
**mE**	**36.06±0.64**	**35.19±0.64**	**-7.30**	**<0.001**	**0.815**	**<0.001**	**0.740**	**0.876**
mL	35.69±0.63	35.00±0.63	-6,33	<0.001	0.785	<0.001	0.707	0.851
Mouth	35.97±0.83	35.15±0.67	-6.22	<0.001	0.783	<0.001	0.704	0.849
Nose	35.56±0.84	35.17±0.76	-2,85	<0,01	0.631	0.009	0.544	0.712
TIC	36.32±0.89	36.11±0.78	-1.51	0.132	0.574	0.138	0.486	0.658

*p-values for means comparisons were obtained from t-student test for independent samples; ^#^p-values for AUC comparisons were obtained from z-test at 95% confidence interval.

**Fig 4:**
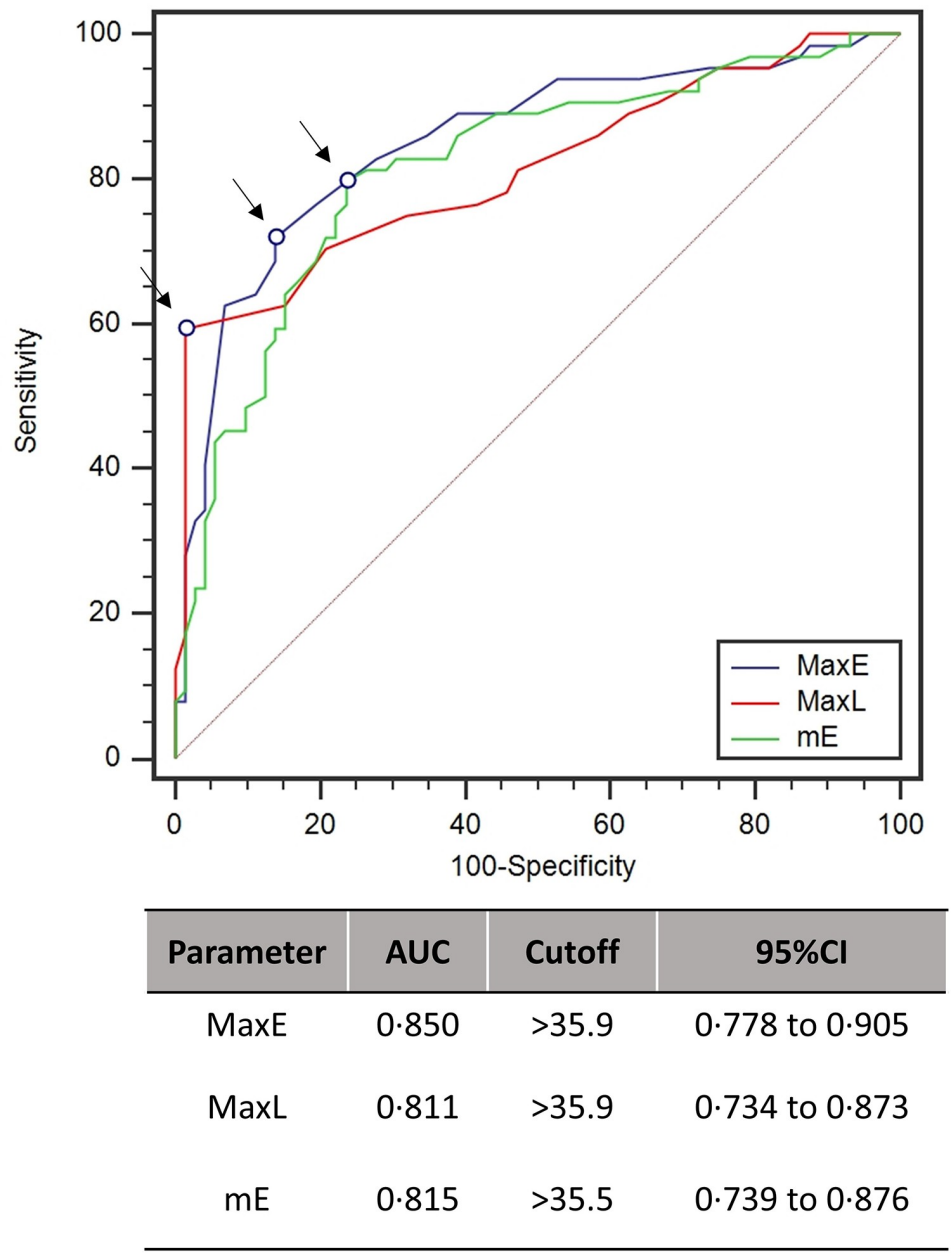
Receiver operator characteristics (ROC) curve with the best IRT parameters. Performance of each parameter is compared through their respective areas under the curve (AUC), 95% confidence intervals are also provided. Arrows indicate the criterion cutoff points. MaxE (maximum temperature of the Eyes), MaxL (maximum temperature of the lateral of the nose), mE (mean temperature between the eyes).

The performance of our best infrared variables obtained in the receiving operating curves is shown in Fig 4: the max and the mean temperature of the eye (MaxE and mE) showed the highest value to discriminate infected from non-infected patients between both sets of parameters (AUC = 0.850, 95%CI: 0.779–0.906, p<0.001 and AUC = 0.815; 95%CI: 0.740–0.876, p<0.001, respectively), followed the max temperature of the lateral portion of the nose (MaxL) (AUC = 0.811; 95%CI: 0.735–0.873, p<0.001). On the other hand, the mean temperature of the TIC (AUC = 0.574, 95%CI: 0.486–0.658, p = 0.13) showed the worst discriminatory power among the IRT variables (Table 2).

The cutoff analysis showed that the best trade-off between sensitivity and specificity for our most discriminant infrared variables were: MaxE: >35.9°C, with a Youden J index of 0.58, an accuracy of 80% (sn = 71.87%, sp = 86.11%, LR+ = 5.18, PPV = 0.766, LR- = 0.33, NPV = 0.829); MaxL: >35.9°C, Youden J index of 0.58, an accuracy of 80% (sn = 59.38, sp = 98.61, LR+ = 42.75, PPV = 0.964, LR- = 0.41, NPV = 0.794) and mE: >35.6, Youden J index of 0.56, accuracy of 78% (sn = 79.69%, sp = 76.39%, LR+ = 3.37, PPV = 0.680, LR- = 0.27, NPV = 0.856) (Table 3).

**Table 3:** Criterion validity for all infrared parameters evaluated in this study. The cutoff values represent the best trade-off between sensitivity and specificity in the 136 patients analyzed in the emergency room of HU-USP Hospital, São Paulo, Brazil.

Variable	Criterion	Sensitivity	95% CI	Specificity	95% CI	LR+	95% CI	LR-	95% CI	PPV	95% CI	NPV	95% CI	YoudenJ
**MaxE**	**>35.9**	**71.87**	**59.2 - 82.4**	**86.11**	**75.9 - 93.1**	**5.18**	**2.9 - 9.4**	**0.33**	**0.2 - 0.5**	**76.6**	**62.4 - 87.4**	**82.9**	**73.3 - 90.1**	**0.58**
**MaxL**	**>35.9**	**59.38**	**46.4 - 71.5**	**98.61**	**92.5 - 100.0**	**42.75**	**6.0 - 302.5**	**0.41**	**0.3 - 0.6**	**96.4**	**83.2 - 99.9**	**79.4**	**70.3 - 86.7**	**0.58**
EyeD	>35.8	70.31	57.6 - 81.1	83.33	72.7 - 91.1	4.22	2.5 - 7.2	0.36	0.2 - 0.5	72.7	58.4 - 84.2	81.6	71.8 - 89.2	0.54
EyeE	>35.7	71.87	59.2 - 82.4	77.78	66.4 - 86.7	3.23	2.0 - 5.1	0.36	0.2 - 0.5	67.1	53.3 - 79.1	81.4	71.1 - 89.3	0.50
**mE**	**>35.5**	**79.69**	**67.8 - 88.7**	**76.39**	**64.9 - 85.6**	**3.37**	**2.2 - 5.2**	**0.27**	**0.2 - 0.4**	**68.1**	**55.0 - 79.4**	**85.6**	**75.6 - 92.7**	**0.56**
LFD	>35.9	42.19	29.9 - 55.2	98.61	92.5 - 100.0	30.38	4.2 - 217.2	0.59	0.5 - 0.7	95.0	77.4 - 99.8	73.0	63.8 - 80.9	0.41
LFE	>35.6	56.25	43.3 - 68.6	83.33	72.7 - 91.1	3.38	1.9 - 5.9	0.53	0.4 - 0.7	68.1	52.2 - 81.4	75.1	65.0 - 83.5	0.40
mL	>35.55	60.94	47.9 - 72.9	86.11	75.9 - 93.1	4.39	2.4 - 8.1	0.45	0.3 - 0.6	73.5	57.9 - 85.7	77.7	67.9 - 85.7	0.47
Mouth	>35.6	71.87	59.2 - 82.4	77.78	66.4 - 86.7	3.23	2.0 - 5.1	0.36	0.2 - 0.5	67.1	53.3 - 79.1	81.4	71.1 - 89.3	0.50
Nose	>35.8	39.06	27.1 - 52.1	81.94	71.1 - 90.0	2.16	1.2 - 3.9	0.74	0.6 - 0.9	57.7	40.1 - 74.1	68.1	58.0 - 77.0	0.21
TIC	>36.1	57.81	44.8 - 70.1	56.94	44.7 - 68.6	1.34	1.0 - 1.9	0.74	0.5 - 1.1	45.9	33.6 - 58.6	68.1	55.9 - 78.8	0.15

Note: LR+: positive likelihood ratio, LR-: negative likelihood ratio, PPV: positive predictive value, NPV: negative predictive value and 95% CI: confidence interval. Values were calculated using a prevalence of 38.7%, registered in the western region of São Paulo [20]. *P<0.001, IC95%.

**Fig 5:**
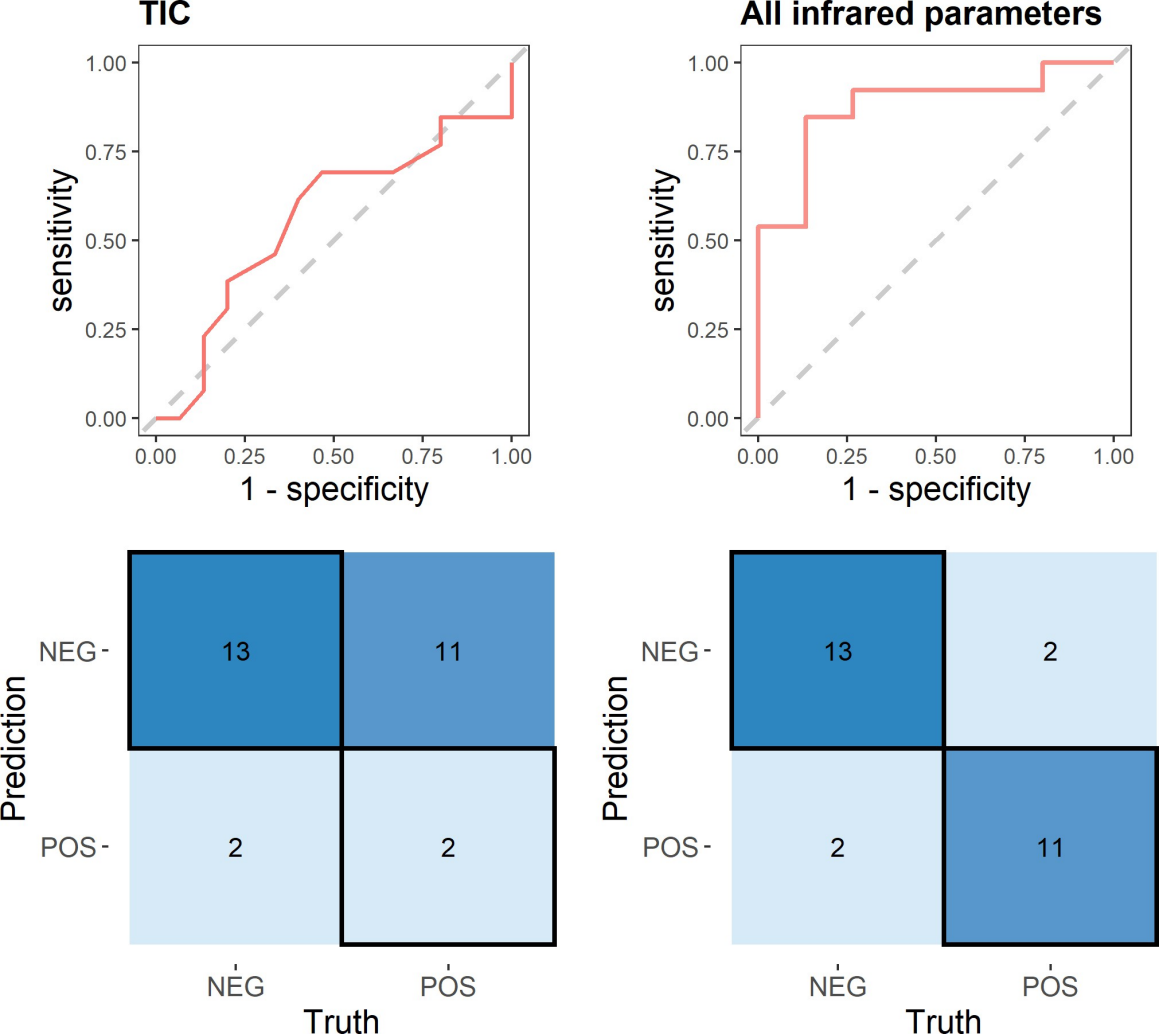
ROC curves and confusion matrix of predictive models for COVID-19. (a) Logistic regression with max temperature of eye inner canthus (TIC) as the only predictor and (b) Random Forest model (mtry = 4, min_n = 3, trees = 1000), combining all thermal parameters as predictors. RF model showed vastly improved performance, evidencing that considering one single temperature spot (common practice in the infrared solutions currently) is problematic for COVID-19 detection.

**Fig 6:**
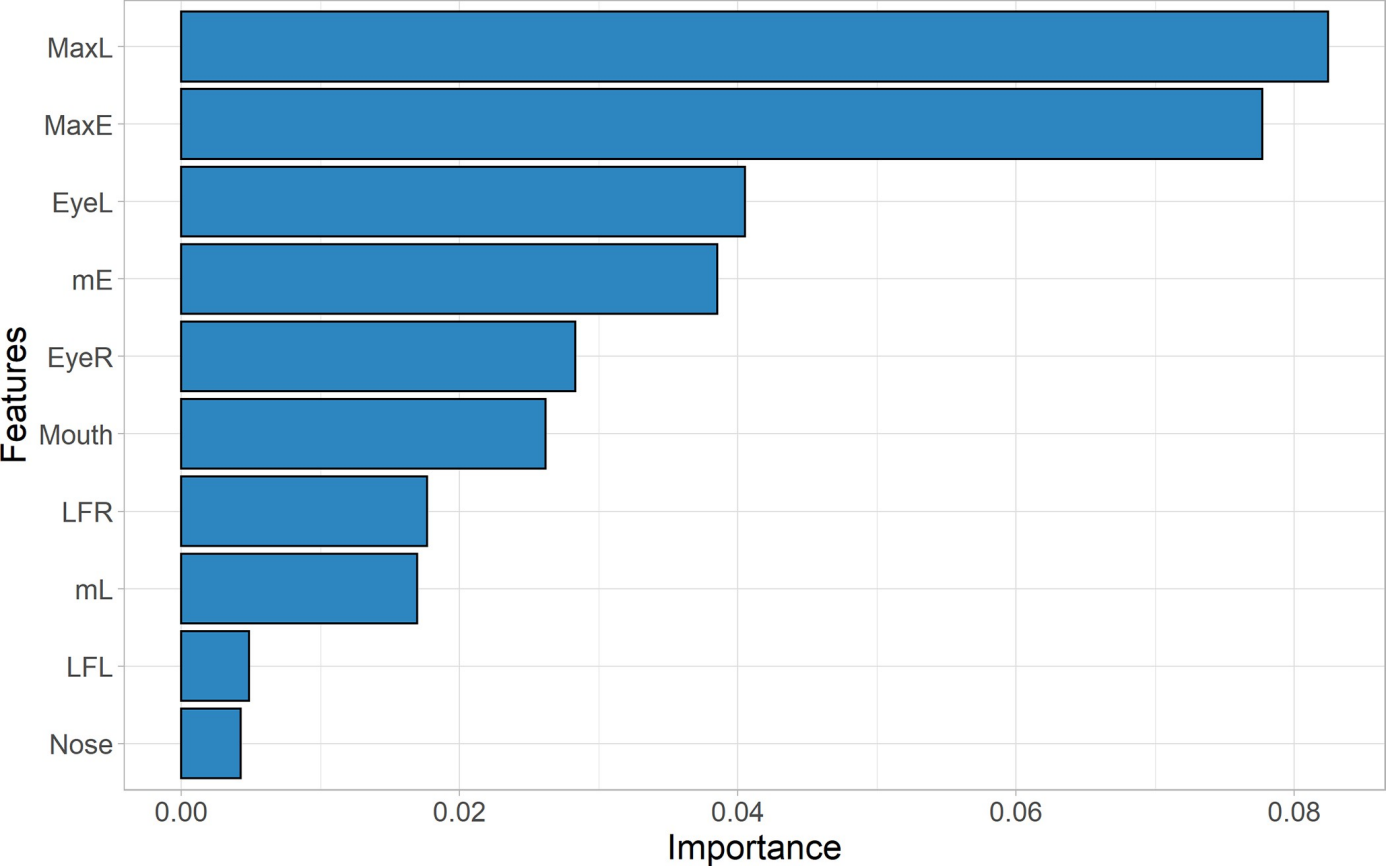
Feature importance analysis based on permutation technique (extracted from the Random Forest Model). The most important features responsible for model classification were MaxL and MaxE.

Finally, our predictive model for COVID-19, with all facial infrared parameters combined, reached an accuracy of 86% [95% CI: 0.67 to 0.96] (Sn = 0.84, 95% CI: 0.55 to 0.98; Sp = 0.86, 95% CI: 0.60 to 0.98 and AUC = 0.87, 95% CI: 0.72 to 0.99), performing dramatically better than when considering a unique facial spot as core temperature estimator, in our case the maximum temperature of eye *inner canthus* (TIC) (acc = 0.53, 95% CI: 0.34 to 0.72; sn = 0.15, 95% CI: 0.02 to 0.45; sp = 0.86, 95% CI: 0.60 to 0.98 and AUC = 0.56, 95% CI: 0.47 to 0.67) (Fig 5). According to Fig 6, the most important features related to model classification were again MaxL and MaxE. Additional information on the model robustness with the resamples metrics are provided in S1 Table.

## 4. Discussion

Among the PCR+ patients, fever (56%), cough (59%), anosmia (52%), and myalgia (44%) were the most common self-reported symptoms (Table 1). Similarly, a study carried out with 20133 patients with suspicion of COVID-19 infection in United Kingdom hospitals reported that the most common symptoms were cough, fever, and shortness of breath and only 4.5% reported no symptoms on admission [21]. Also, the most observed symptoms in 138 patients monitored in a study performed in Wuhan, were fever, fatigue, dry cough, myalgia, and dyspnea [22]. Another study, conducted in Italy with 143 patients, showed that fatigue, dyspnea, cough, and myalgia were the most common during the acute phase of infection [23]. Although Diarrhea/Emesis were the least common symptoms reported by our patients, its occurrence was significantly higher in infected patients (28% in positive cases versus 7% in negative, p < 0.01). In fact, gastrointestinal dysfunctions were reported by several authors as a COVID-19 symptom and they may be related to enterocyte absorption disorder and/or intrahepatic bile ducts injury [10, 21–24].

Fever is the most common clinical finding regarding COVID-19, leading to health centers, offices, and airports to set up screening centers to assess this particular parameter using IRT [6]. The temperature of the forehead is usually adopted as the main parameter to assess fever through IRT, however, its usage implies critical limitations, such as interference of ambient temperature, alcohol or food consumption, sunburn, and other skin conditions and may not reflect core body temperature (T*core*) [7]. In an extensive study of fever clinical evaluation, Zhou et al. [10] analyzed different facial locations and confirmed the utility of eye inner canthus for fever screening. Furthermore, the author also concluded that the full-face maximum temperature may also be useful as an alternate approach. The hole of the eye inner canthus as the most efficient infrared fever screening or Tcore estimator is unchallenged in this paper, since our data confirmed that, for the most cases, it indeed reflected the maximum facial temperature among all regions evaluated. Some studies reported fever as a common symptom of COVID-19 [6, 25–27], however, we found no direct association between febrile status and infected patients; only 19% of infected patients presented fever in the hospital evaluation. In fact, other studies challenged that fever itself would be a good parameter for screening for COVID-19, requiring caution to be used for screening. A study conducted in an Australian hospital found that fever was also a negligible indicator for detection of COVID-19, presenting as a symptom in only 19% episodes of positive tests for the disease [6]. Similar results were found in a study with patients between 18 and 25 years old, the authors concluded that screening for fever is not sensible enough to detect the majority of COVID-19 cases in that age range, due to fever being highly episodic and detected only in the first few days of infection [28]. Furthermore, a cut-off of 38.5°C missed 92% of coronavirus cases and even a low-temperature cut-off of 37.1°C would miss a third of the cases [28]. The problem here is not that infrared thermography, throughout the temperature of eye inner canthus, would not provide a reliable measurement for fever estimation, but the symptom itself may not be a reliable parameter in certain situations.

Accordingly, our results showed that temperature estimation based on one single spot (TIC), was the worst parameter to discriminate between infected and non-infected with SARS-CoV-2 virus; a cut-off of 37.6°C was not sensible enough to detect the most cases of the disease (Sn = 12.5%), suggesting that this parameter may be not adequate to screen for COVID-19 (Table 3). A recent study showed that SARS-CoV-2 replicates to higher tilters at a low temperature of 33°C, characteristic of the upper respiratory tract, while SARS-CoV replicates more efficiently at 37°C, in the lower respiratory tract [29]. Such findings reinforce the negligible hole of fever to differentiate infected from non-infected with COVID-19 and reiterate why caution must take place when this particular parameter is being used solely to screen for the novel coronavirus. Once the face is highly vascularized and remarkably sensitive to subtle endothelial temperature changes, it is easier to detect early signs of inflammations caused by infectious diseases in the upper respiratory tract [11]. Thus, thermal facial profile (Fig 2), as used in this study, produces a clearer picture of signs of inflammation of upper airways, providing important symptomatic clues that may be associated with COVID-19 (and other SARs syndromes), rather than only evaluating T*core*.

In fact, we found that three facial IRT parameters (MaxE, MaxL, and mE) showed the highest potential to work as a symptom evaluator for COVID-19 and thus providing a good screening value for this disease; two of them (MaxE and mE) being associated with the eye’s region (Table 3). The ocular manifestation of COVID-19 has been the subject of recent studies and could explain the pattern we found. Some studies indicated that SARS-CoV-2 virus may be detected in the tears and the conjunctival secretions of infected patients with conjunctivitis [30], however other authors found no evidence of the virus presence in eye secretions [31]. Although it is still unclear whether or not this virus can be transmitted through tears or conjunctivitis secretions, symptoms related to eye impairment have been reported for the novel coronavirus [32]. In fact, it was observed that one-third of patients infected with coronavirus presented ocular abnormalities, such as chemosis, epiphora, and conjunctival hyperemia, being more frequent in patients with the more severe manifestations of the disease in Hubei Province, in China [33]. Moreover, around 30% of 143 infected patients in an Italian hospital presented red eyes as symptoms of COVID-19, indicating eye impairment to some extent [23]. In 2020, a meta-analysis was conducted and the authors concluded that conjunctivitis may represent a sign of COVID-19 infection associated to more severe form of the disease [34]. The eye limbus was found to be the most susceptible region to SARS-CoV-2 infection, mounting a significant inflammatory response [32]. In fact, the unique study associating IRT imaging of face to COVID-19 screening potential found promissory results: the authors found that cutoff values of ≥ 0.55 between the temperatures of forehead and eye caruncle are highly suggestive of COVID-19 infection [11]. More importantly is that the extraction of the thermal profiles allows the detection of signs of eye inflammation or alterations in the tear film [35, 36] that may be also related to other diseases, such as, COVID-19 or other SARs syndromes.

Regarding the criterion validity of IRT parameters, both MaxE and MaxL performed better as COVID-19 predictors at a cut-off of >35.9°C, with 80% accuracy, followed by the mean temperature of the eyes (mE) with a best cut-off scenario was at >35.5°C and an accuracy of 78% (Table 3). Interesting to note that none of the cutoff values lied within fever state, emphasizing that the latter vital signal has a poor value to screen for the novel coronavirus. Thus, thermal asymmetries among MaxE, MaxL, and mE should be prioritized by on-duty health personnel to screen for COVID-19, while using IRT face imaging mapping.

Our predictive model, based on the combination of all facial thermal parameters was vastly superior on positive detection (acc = 86%, sn = 0.84) when compared to the standard procedures using on single spot representing the maximum face temperature and thus an estimate of body temperature (acc = 0.53, sn = 0.15) (Fig 5). However, is not the objective of this paper to propose a definitive diagnostic test for COVID-19 based on thermography, but show how this technology can be used in a meaningful way to improve the quality of currently infrared techniques commonly employed in screening procedures, **being used as additional and more accurate tool for diagnosis support of flu syndromes**

In sum, our findings demonstrated that a solution, based on thermal asymmetries of the facial thermal profile, combining different areas, is more accurate to screen for COVID-19, when compared to the most common infrared procedure available today, based on a punctual facial temperature measure to assess fever.

Although the sample size is relatively small and the characteristics of the patients are homogenous, which complicates generalizations, a broader understanding of the pandemic situation in Brazil is needed to be put in context. At the time of data collection RT-qPCR SARS-CoV-2 kits were scarce and expensive and only patients with severe symptoms of SARS were eligible to be tested in the hospital, according to government guidelines. This could lead to potential selection bias of our sample. Still, we collected data of almost all patients that sought help in the hospital during the period of collection.

Furthermore, the prevalence of the disease used in this study does not reflect the actual values in Brazil, since the pandemic situation has altered since data collection and underreporting of cases is still noticeable in the country, compromising the calculations of the predictive values of our test. Therefore, the PPV/NPV values cannot be extrapolated to other samples, since the prevalence of the disease may vary widely among different regions of the country. The fact that the sample is limited to a specific location and socioeconomic condition could also affect the predictive power of our IRT parameters.

In conclusion, the results of the present study suggest that face thermography is applicable in diagnostic support for flu-like syndromes and proved to be quite efficient during the beginning of the COVID-19 pandemic in the emergency center of the University Hospital of USP. The parameters related to the eyes and lateral portions of the nose (MaxE, mE and MaxL) showed a good potential to discriminate infect from noninfected subjects, indicating signs of early inflammation of the upper airways, typical in SARs syndromes, such as COVID-19. Furthermore, we showed that the predictive power using the facial thermal profile was vastly superior when compared to only one facial temperature spot (TIC) (Fig 5). Infrared thermography is an image technique that has unique advantages as it requires no physical contact and produces no harmful radiation, has high reproducibility, repeatability and a fast turnaround. These characteristics, coupled with its potential to detect early signs of inflammation, qualifies this technology as a good candidate for enhancing screening procedures in hospitals and health care centers to evaluate early signs of infectious diseases, as shown in this study.

## Supporting information

S1 Table Result of cross validation through bootstrap resampling (K = 25) for the Random Forest classification model. (DOCX)

S1 Fig ROC curves for cross validation resamples (K = 25) for the Random Forest classification model. (TIF)

S2 Fig Results of hyperparameter tuning for the Random Forest classification model (best results: mtry = 4 and min_n = 3). (TIF)

S1 Data Raw data. (XLSX)

S2 Data Random Forest code. (RMD)

## Abbreviation

IRT: Infrared thermography
TIC: max temperature of eyes inner canthi
MaxE: max temperature between both eyes
MaxL: max temperature between both sides of the nose
EyeR: maximum temperature of right eye surface
EyeL: max temperature of left eye surface
LFR: max temperature of the right lateral portion of the nose surface
LFL: max temperature of left lateral of the nose
mE: mean temperature of both eyes
mL: mean temperature between both lateral portions of the nose
Mouth: mean temperature of the mouth
Nose: mean temperature of the nose surface
acc: accuracy
sn: sensitivity
sp: specificity
AUC: area under the curve
