# Supplementary material for: Potential of using facial thermal imaging in patient triage of flu-like syndrome during the COVID-19 pandemic crisis
Source: PLoS One. 2023 Jan 18;18(1):e0279930. doi: 10.1371/journal.pone.0279930 (PMC9847904; doi:10.1371/journal.pone.0279930)
Supplement: S1 Table — (DOCX) [file pone.0279930.s001.docx]

**S1 Table. Result of cross validation through bootstrap resampling (K=25) for the Random Forest classification model.**

| **Metric** | **Estimator** | **Mean** | **Std_err** | **n (resamples)** |
| --- | --- | --- | --- | --- |
| Accuracy | binary | 0.802 | 0.011 | 25 |
| AUC | binary | 0.900 | 0.008 | 25 |
| Sensitivity | binary | 0.753 | 0.020 | 25 |
| Specificity | binary | 0.847 | 0.016 | 25 |
