# Supplementary figures and images for: Potential of using facial thermal imaging in patient triage of flu-like syndrome during the COVID-19 pandemic crisis
Source: PLoS One. 2023 Jan 18;18(1):e0279930. doi: 10.1371/journal.pone.0279930 (PMC9847904; doi:10.1371/journal.pone.0279930)

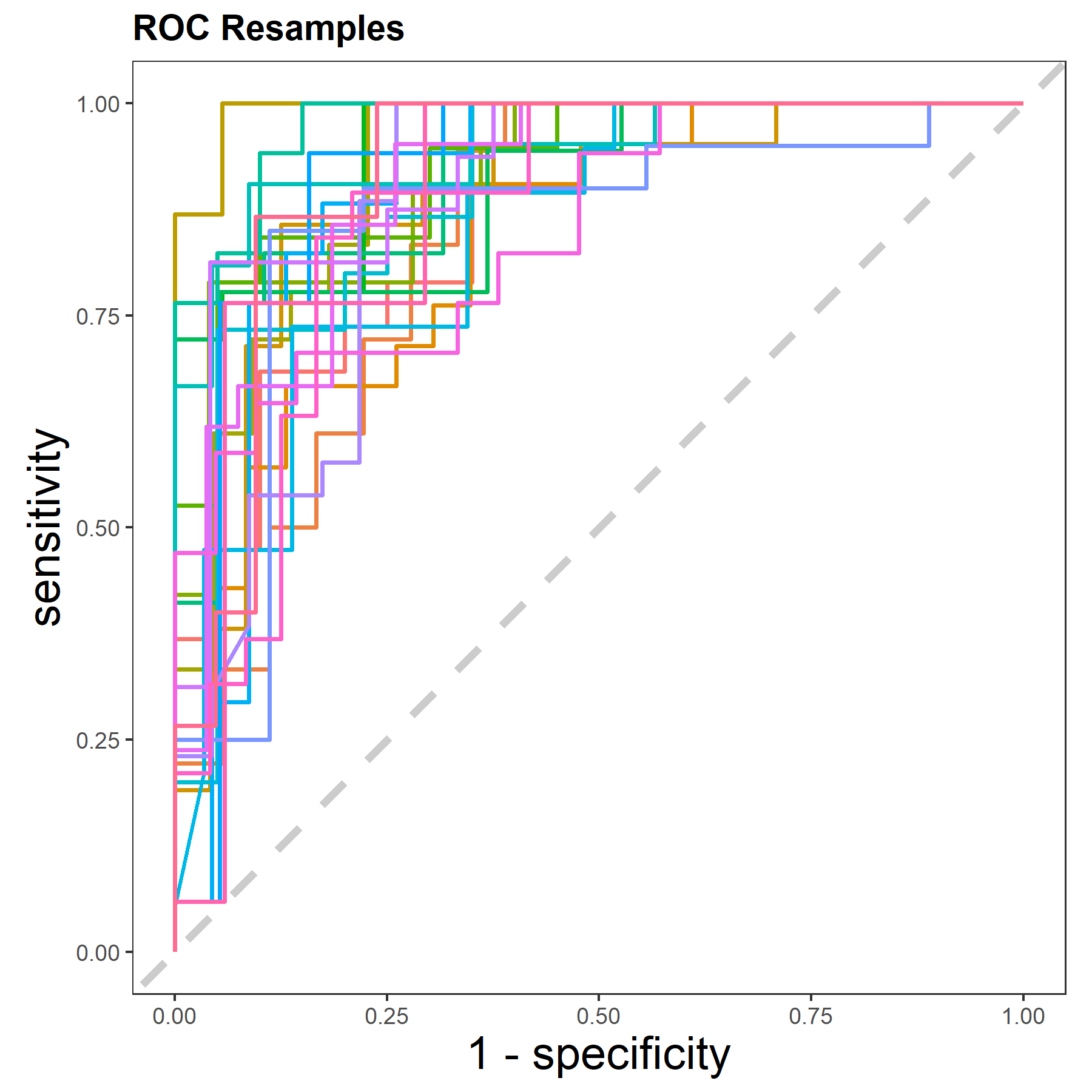

Supplement: S1 Fig — (TIF) [file pone.0279930.s002.tif]

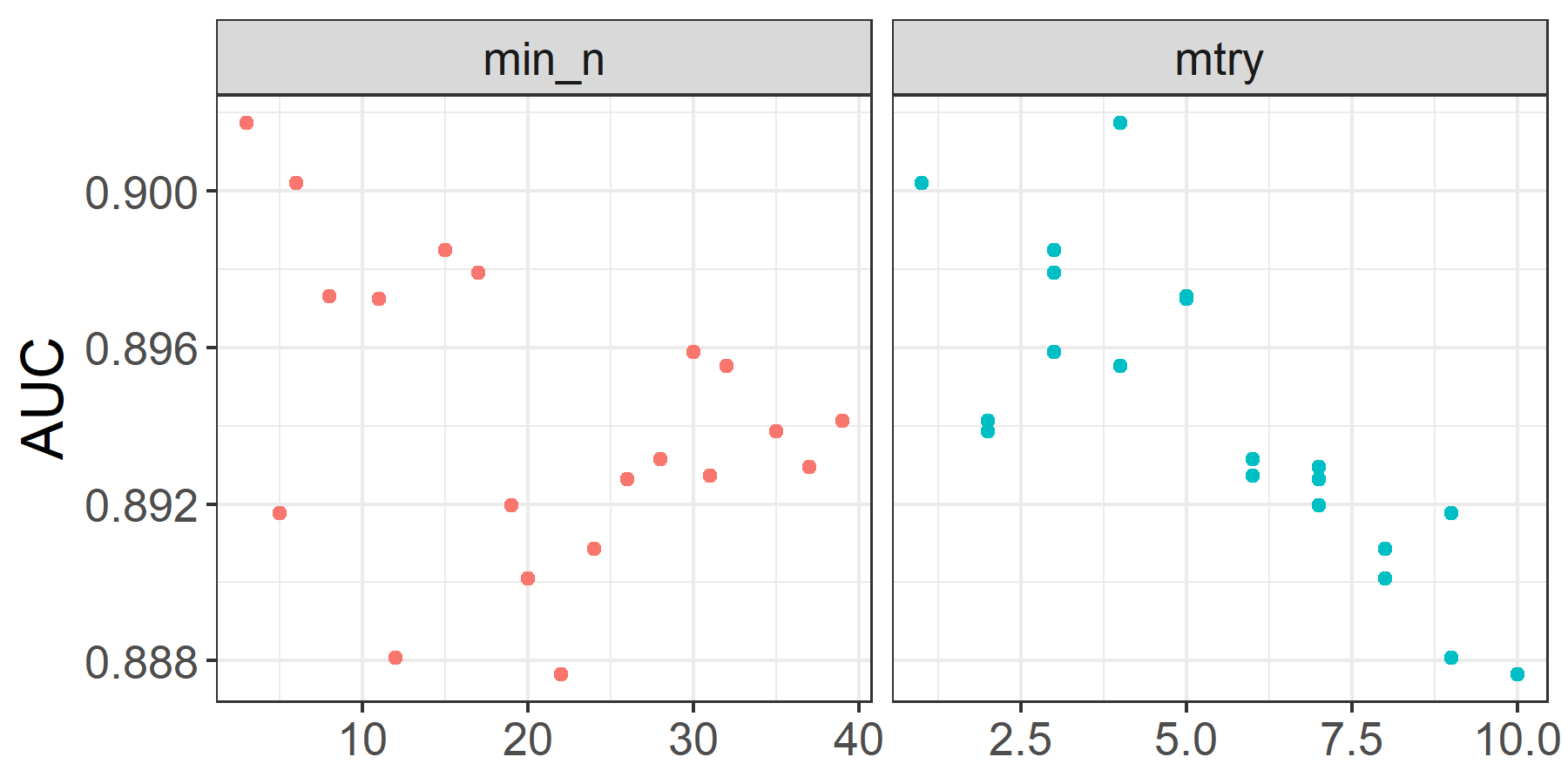

Supplement: S2 Fig — (TIF) [file pone.0279930.s003.tif]
